# Cost-effectiveness analysis of universal newborn screening for medium chain acyl-CoA dehydrogenase deficiency in France

**DOI:** 10.1186/1471-2431-12-60

**Published:** 2012-06-08

**Authors:** Françoise F Hamers, Catherine Rumeau-Pichon

**Affiliations:** 1 Department of Economic and Public Health Evaluation, Haute Autorité de Santé (HAS), 2 avenue du Stade de France, Saint-Denis, France

**Keywords:** Medium-chain Acyl-CoA dehydrogenase deficiency, Cost effectiveness, Neonatal screening, Health policy, Tandem mass spectrometry, France

## Abstract

**Background:**

Five diseases are currently screened on dried blood spots in France through the national newborn screening programme. Tandem mass spectrometry (MS/MS) is a technology that is increasingly used to screen newborns for an increasing number of hereditary metabolic diseases. Medium chain acyl-CoA dehydrogenase deficiency (MCADD) is among these diseases. We sought to evaluate the cost-effectiveness of introducing MCADD screening in France.

**Methods:**

We developed a decision model to evaluate, from a societal perspective and a lifetime horizon, the cost-effectiveness of expanding the French newborn screening programme to include MCADD. Published and, where available, routine data sources were used. Both costs and health consequences were discounted at an annual rate of 4%. The model was applied to a French birth cohort. One-way sensitivity analyses and worst-case scenario simulation were performed.

**Results:**

We estimate that MCADD newborn screening in France would prevent each year five deaths and the occurrence of neurological sequelae in two children under 5 years, resulting in a gain of 128 life years or 138 quality-adjusted life years (QALY). The incremental cost per year is estimated at €2.5 million, down to €1 million if this expansion is combined with a replacement of the technology currently used for phenylketonuria screening by MS/MS. The resulting incremental cost-effectiveness ratio (ICER) is estimated at €7 580/QALY. Sensitivity analyses indicate that while the results are robust to variations in the parameters, the model is most sensitive to the cost of neurological sequelae, MCADD prevalence, screening effectiveness and screening test cost. The worst-case scenario suggests an ICER of €72 000/QALY gained.

**Conclusions:**

Although France has not defined any threshold for judging whether the implementation of a health intervention is an efficient allocation of public resources, we conclude that the expansion of the French newborn screening programme to MCADD would appear to be cost-effective. The results of this analysis have been used to produce recommendations for the introduction of universal newborn screening for MCADD in France.

## Background

Medium chain acyl-CoA dehydrogenase deficiency (MCADD) is a hereditary metabolic disease characterised by decreased ability of the body to use fat as a source of energy during periods of fasting or increased metabolic need. It is due to a deficit of the medium chain acyl-CoA dehydrogenase enzyme and is transmitted through an autosomal recessive mode. Affected individuals may present with hypoketotic hypoglycaemia, which may lead to coma or death. If individuals are detected before a life-threatening episode, the complications of MCADD are, however, preventable by avoiding fasting stress and providing regular feeds in the first years of life.

The prevalence of MCADD at birth among Caucasian populations ranges between 1/10 000 and 1/27 000 [1-8]. In France, while epidemiological studies of a sufficient sample size have not been conducted, it is nevertheless likely that the prevalence lies within the range of the extreme values found in the neighbouring countries.

Universal newborn screening based on a dried blood spot test is a well-established, government-funded programme in France. It is currently organised through a network of 22 regional labs and its coverage is over 99.99%. It includes five diseases – phenylketonuria (PKU), congenital hypothyroidism, congenital adrenal hyperplasia, cystic fibrosis, and, among high-risk populations, sickle cell disease.

The development of tandem mass spectrometry (MS/MS) in the early 1990s led to a substantial increase in the number of potentially detectable hereditary metabolic diseases. This technology is being used to screen newborns for an increasing number of diseases in an increasing number of countries in Europe [9] and elsewhere.

The French National Authority for Health (HAS) was asked by the Ministry of Health to evaluate options and to produce public health recommendations concerning the expansion of the national newborn screening programme for inborn errors of metabolisms using MS/MS. Based on a preliminary literature review, it was agreed to start by evaluating the expansion of newborn screening to MCADD, a disease for which there is ample evidence to suggest that newborn screening is an effective and cost-effective intervention [10-22]. Cost-effectiveness was considered by the French health authorities to be an important element to inform policy decision even though France has not defined any incremental cost-effectiveness ratio (ICER) threshold for the implementation of new public health interventions.

Several economic analyses of MCADD newborn screening have been performed in Europe [10,13-15] and North America [18-23], and several reviews of such economic analyses have been published [10,13,24,25]. Estimates of cost-effectiveness of MCADD screening varied widely, depending on the modelling assumptions [25].

One Canadian study estimated an ICER of 253 161 Canadian dollars (about €200 000) per life year (LY) [22], assuming systematic lifetime supplementation of carnitine. If, however, it was assumed that supplements were provided up to the age of 5 years only, MCADD screening became cost-saving. In another Canadian study [21], where no carnitine supplementation was assumed, the ICER was estimated to be 2 676 Canadian dollars (about €2 000) per quality-adjusted life year (QALY). As highlighted in a recent review, more data are needed to reduce the uncertainty surrounding a number of parameters, particularly the proportion of MCADD cases who die in the first few days of life and will thus never be detected by screening, the effectiveness of screening in preventing MCADD deaths, the quality of life attached to the different health states, and the costs of diagnosis and treatment in the absence of screening [25]. Because of health system specificities and limitations in transposing health care costs from one country to another, it was felt important to carry out a cost-effectiveness analysis of MCADD newborn screening, taking into account the French setting and, where available, using local data.

## Methods

### Model structure, data sources and sensitivity analyses

We developed a decision model to evaluate the cost-effectiveness of the expansion of newborn screening for MCADD in France. We used the decision analysis software TreeAge Pro 2009 Healthcare (TreeAge Software Inc, Williamstown, MA, USA). The model was structured according to the economic evaluation methodological choices defined by HAS [26]. We used a societal perspective and a lifetime analytic time horizon, expressed the health consequences in QALY, and used an annual discounting rate of 4% for both costs and health effects.

Because PKU, but not the other conditions currently screened for in France, can be detected by MS/MS, the expansion of newborn screening to MCADD was evaluated alongside a concurrent switch in the technology used for PKU screening, from the current fluorometric method, to MS/MS. The economic modelling was performed in two steps.

**Figure 1:**
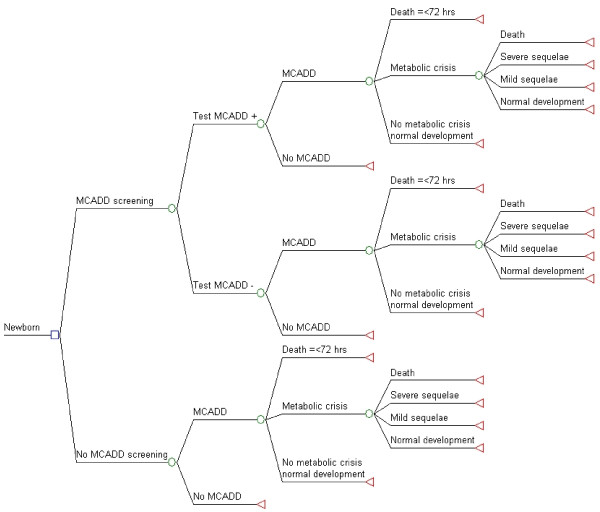
**Decision tree: Expansion of newborn screening to include MCADD*vs.*current newborn screening.** The square represents a decision node, circles chance nodes, and triangles terminal nodes. The branch of the decision node “MCADD screening” is compared to “No MCADD screening” which is the current situation. Probabilities, costs, and QALY are calculated at each terminal node according to the parameters described in Table 1. To evaluate the concurrent cost-effectiveness of switching to MS/MS for PKU screening, the cost of the current technology to screen for PKU was subtracted from the cost of the MCADD screening test as described in the text.

In the first step, we evaluated the consequences of expanding the existing newborn screening program to include MCADD screening (Figure1), which implies the introduction of the MS/MS technology. Second, we evaluated the consequences of replacing the fluorometric method currently used for PKU with MS/MS. In this second step, we assumed, conservatively, that the performances of the PKU screening test were identical for both technologies, which implies that the health outcomes were also identical, regardless of the screening technology used. As the cost of MS/MS screening remains virtually unchanged irrespective of the number of conditions being screened, the consequences of this second step only pertain to the incremental cost of the MS/MS screening test over and above the cost (no longer incurred) of performing fluorometry for PKU.

Health effects were expressed in LY and in QALY gained. We calculated, for a French birth cohort (821 000 live births), the incremental cost/saving of screening and early identification of MCADD (step 1) and of using MS/MS for PKU screening (step 2); the incremental health effect in terms of additional LY gained and corresponding QALY gained; and the ICER, expressed in € per LY and per QALY gained.

(1) $$ICER=\frac{Incremental\;cost/saving\;MCADD\;screening-cost\;PKU\;fluorometry\;test}{QALY(or\;LY)gained}$$

Parameter values were based on published and routine data sources, where available; otherwise, expert judgment was used (Table 1). Systematic reviews of the literature were conducted using various electronic searches (MEDLINE, EMBASE), government reports, and hand searching journals and reference lists on the following topics: MCADD screening tests, diagnosis, outcome, treatment, effectiveness, cost, and cost-effectiveness. We used French administrative databases to estimate costs of MCADD sequelae. We used the national health insurance 2010 official rate for specialized medical consultation (€ 23 per consultation). Costs of MCADD tests were estimated based on 2010 commercial prices of equipment and reagents, and salaries, in consultation with the national screening programme. Details on the search strategies and on the values and plausible ranges for the model’s parameters and the calculations performed are available (in French) at http://www.has-sante.fr/portail/upload/docs/application/pdf/2011-07/argu_depistage_neonatal_vf.pdf [27].

**Table 1:** Value of the parameters used in the MCADD newborn screening cost-effectiveness analysis model

**Parameter**	**Base-case value**	**Range for sensitivity analyse**s	**References**
**MCADD birth prevalence**	1/15 000	1/10 000–1/25 000	Expert judgement^*^
**Performances of MCADD screening test**			
Sensitivity	1		[28]
Specificity	0.9998	0.9997 – 1	[2,4,29-36]
**Probability of MCADD complications**			
Death ≤ 72 hours of life	0.02	0.02 – 0.05^†^	Expert judgement^*^
Metabolic crisis	0.67	0.67 – 0.75	[37]
Death after a metabolic crisis	0.20	0.10 – 0.30	^‡^[17,38-44]
Severe sequelae^§^	0.05	0 – 0.05	[38-41,43-45]
Mild sequelae§	0.05	0 – 0.05	[38-41,43-45]
**Effectiveness of screening to prevent metabolic crisis**	0.75	0.50 – 0.75	[17]
**Life expectancy (years)**			
Normal	81		French census
Death after metabolic crisis	1.2		[46]
Severe sequelae	56		[47] See text
Mild sequelae	70		[47] See text
**Heath-related quality of life**			
Not affected by MCADD	1	0.90 – 1	See text
Mild sequelae	0.89	0.89 – 0.92	[14]
Severe sequelae	0.76	0.50 – 0.76	[14]
**Costs of screening test**			
Screening test (€) (tests/lab/year)^**^	3.75 (50 000)	3.38 – 5.16 (60 000 – 30 000)	See text
Confirmatory test (€)	500		See text
**Cost of treatment of uncomplicated MCADD and MCADD sequelae**			
L-carnitine (€)^††^	6 065	0 – 12 130	See text
Medical consultations (€)^‡‡^	888	888 – 1 264	See text
Metabolic crisis (€)	2 770	2 770 – 4 730	Database
Severe sequelae^§§^	21 000	15 000 – 150 000	Database
Mild sequelae^§§^	6 000	4 500 – 120 000	Database

^*^Expert judgment based on available literature, see text.

^†^ Mortality within 24 hours of life is susceptible to be lower in the presence (2%) than in the absence (5%) of a screening programme because of better knowledge and awareness of the disease by clinicians.

^‡^ Point estimate produced by pooling available literature data.

§ Neurological sequelae after a metabolic crisis.

^**^ The unit cost of the screening test depends on the annual number of tests per lab (shown in parentheses).

†† Cost of supplement in L-carnitine until the age of 18 shown, discounted. The proportion of patients treated is 50% in the base-case, ranging from 0% to 100% in the alternative scenarios.

‡‡ Cost of medical consultations discounted during the duration of life. The number of medical consultations per year in the absence of complication is two in the base-case analysis and five until the age of 6 then two during the remaining life in the alternative scenario.

§§ Annual cost includes special education and residential care. Lifetime costs were computed based on estimated life expectancies, see text.

One-way sensitivity analyses were conducted on most parameters, which were varied over a range of plausible values to estimate the impact of uncertainty in the data and the robustness of results. A worst-case scenario, where all the parameters that were varied in the one-way sensitivity analyses were set at their most unfavourable values, was also simulated. We also conducted sensitivity analyses on the discount rate for the following values: 0%, 3%, and 6%.

### Probabilities

#### MCADD prevalence

In the absence of any relevant epidemiological study, the prevalence of MCADD in France was assumed to be within the range of extreme values estimated in neighbouring countries. A value of 1 in 15 000 live births was assumed, with a range of plausible values between 1 in 10 000 and 1 in 25 000 [1-8].

#### Screening test performance

MS/MS is the only available method to screen for MCADD. Its specificity, which has been evaluated in a number of studies, ranges from 99.97% to 100% [2,4,29-36]. We used a median value of 99.98%. Its sensitivity is considered to be 100% [28].

The current method used in France to screen for PKU is fluorometry. Its specificity has been estimated to be 99.97% and its sensitivity to be 99.3% [48]. Compared to fluorometry, MS/MS appears to have a greater specificity and sensitivity [49-51]. We made the conservative assumption that these measures were identical for the two methods.

#### Probability of MCADD complications

The probability of dying within 24 hours of life, i.e. before screening could be performed or its results be available, was estimated at 2%. This estimate was based on the results from an Australian study where the proportion of deaths before 24 hours was 2% (1/41) among screened children and 8% (3/30) among unscreened children [52] and from a study in New England where no early neonatal death was observed among 47 children detected with MCADD by screening [35].

In the absence of screening, two thirds to three quarters of individuals with MCADD will develop a metabolic crisis [17,37]. We used a conservative value of two-thirds (67%) for this parameter. The risk of death following a metabolic crisis was estimated at 20% by pooling data from available studies [17,38-44]. Based on available literature, the probability of developing severe and the probability of developing mild neurological complications after a metabolic crisis were both estimated to be 5% [38-41,43-45].

#### Effectiveness of MCADD screening

In a large Australian cohort study on MCADD screening [17], the cumulative relative risk of death or severe metabolic decompensation by age 2 years was estimated to be 0.26 (which would correspond to an effectiveness to prevent adverse outcomes of 74%), with more liberal and more conservative estimates of 0.19 and 0.44, respectively. On this basis, the effectiveness of MCADD screening to prevent a metabolic crisis was assumed to be 75% with a plausible lower-bound at 50%.

### Life expectancies

We assumed that the life expectancy (LE) at birth of a child with MCADD who will not develop neurological sequelae (or otherwise die from MCADD) is the same as that of an average person, i.e. 81 years (the mean between male and female LE in France in 2009) [53]. The LE of a child who will die from an MCADD metabolic crisis was assumed to be 1.2 years [46]. Likewise, the age at which a metabolic crisis occurs was assumed to be 1.2 years.

No data on LE of patients with MCADD and neurological sequelae were identified. Estimates were therefore derived from a population-based study using data from the state of California [47], which calculated a LE of 65 years for children with mild or moderate mental retardation who did not have Down syndrome, and of 51 years for those with severe mental retardation. These figures correspond to reductions of 11 years and 25 years respectively with respect to the average LE of 76 years in the 1992 US life tables used in the Californian study. For the purpose of our model, we subtracted these same figures from the current French LE of 81 years, giving estimated LE of 70 and 56 years, which we used for mild and for severe neurological sequelae, respectively.

### Health-related qualities of life

Quality of life was estimated by a utility score ranging from 0 (death) to 1 (perfect health). We assumed that the utility score of persons with MCADD who did not develop neurological sequelae was the same as that of persons without MCADD and that it was equal to 1. We used a value of 0.9 in the sensitivity analysis to take into account the fact that these persons may suffer from other types of disease or impairment.

We did not reduce the utility score in case of a false-positive screening result, which is defined as an abnormal screening test followed by a repeat screening test or further investigation that does not indicate an MCADD diagnosis. Indeed, a US study suggests that parents have a high tolerance to false positive newborn screening results [54] and the incorporation of the loss in quality of life associated with false positive test results did not noticeably increase the ICER [23].

For the quality of life related to neurological sequelae, only one study, conducted in Finland and based on a multi-attribute utility instrument, estimated utility scores for neurological sequelae due to MCADD, which were 0.89 and 0.76 for mild and severe neurological sequelae, respectively [14].

### Costs

#### Cost of screening test and of diagnostic test

The collection and transportation of dried blood spots are part of the current newborn screening programme. Therefore these costs could be excluded from the cost- effectiveness analysis. Furthermore, any potential changes in the lab location (see below) would have no impact on transportation costs as dried blood spots are sent by post with charges that are uniform across France.

Therefore, the cost of the MCADD screening test used in the model was limited to lab costs including capital equipment, consumables and personnel. Costs of equipment, including maintenance and consumables, were provided by screening lab experts and also obtained from suppliers. Personnel inputs required to operate MS/MS in a newborn screening lab were estimated in consultation with the national newborn screening programme. The cost of a MS/MS machine represents a high initial capital spending. It constitutes a fixed cost regardless of the number of specimens screened, at least for a large range of number of specimens up to the maximum capacity of the machine.

The introduction of MS/MS will most likely imply a reduction in the number of labs that are performing newborn screening tests, in order to guarantee the quality of the technical expertise and to achieve efficiency. On the basis of 50 000 specimens tested per machine per year and a machine lifespan of 5 years, and using a 4% annual discount rate, the cost of MCADD screening was estimated to be €3.75 per test. The estimated cost of the screening test decreased at a decreasing rate with the number of specimens tested per machine (and hence per lab, assuming one machine per lab) per year, from €5.85 for 25 000 specimens to €2.67 for 100 000 specimens per machine per year.

The cost of the diagnostic test to confirm a positive screening test depends on the screening algorithm used. It was estimated in consultation with the national screening programme to be €500, which includes the costs of contacting the child, of a medical consultation and of additional lab tests. It was assumed, conservatively, that both the cost of further investigation in case of a false positive screening test result and the cost of diagnosing a case of MCADD, in the absence of screening, would also be €500.

Based on the fee paid by the national health insurance scheme to the screening labs [55], the cost of the existing technology for PKU screening (fluorometry) was estimated at €1.76 per test.

#### Cost of uncomplicated MCADD

The management of uncomplicated MCADD consists in dietary management to preventing fasting stress. It was assumed, based on expert opinion, that persons diagnosed with MCADD have on average two medical consultations (at €23 each) per year until the age of 6 years, then one consultation per year for the rest of their life. In a sensitivity analysis, the number of medical consultations was varied from two to five per year during the first 6 years of life. Regarding L-carnitine supplementation, there is no good quality study nor expert consensus on its effectiveness [10,56], data on prescription habits in France are lacking, and its use in other countries is heterogeneous. Therefore, in the base-case analysis, it was assumed that 50% of children diagnosed with MCAD receive a supplementation in L-carnitine until the age of 18 years, after which the supplementation is stopped. Two alternative scenarios were evaluated in the sensitivity analysis where, respectively, no children and all diagnosed children receive L-carnitine until the age of 18 years. Costs were estimated on the basis of the recommended posology and mean weights for age according to French growth charts. In the reference scenario, the discounted cost, for a population LE of 81 years, was estimated at €5 831 (€888 for the medical consultations plus €4 942 for L-carnitine supplementation).

#### Cost of MCADD complications and sequelae

The cost of a metabolic crisis was estimated using the hospital administrative database “Programme de médicalisation des systèmes d’information” (PMSI) by computing the weighted average fee of the disease related groups corresponding to the International Classification of Disease (ICD) 10 code “disorders of fatty-acid metabolism”. The cost amounted to €2 770.

There are no specific data available on the costs of managing neurological sequelae of metabolic diseases in France. Using a French claim database (“Echantillon généraliste des bénéficiaires”), we analysed the annual management cost for patients with long-term illnesses diagnosed with mental retardation (ICD-10: F70–79), as a proxy for severe MCADD neurological sequelae, and for patients with behavioural and emotional disorders with onset in childhood and adolescence (ICD-10: F90–98) as proxy for mild sequelae. These costs, which are covered by the national health insurance scheme, include health care costs as well as other expenses such as special education, residential care, and transportation. They were highly variable, with a median of €21 352 (99% confidence interval [CI]: €17 074–€25 540) and a mean of €125 106 (99% CI: €101 450–€148 171) for mental retardation and a median of €6 306 (99% CI: €4 822–€8 703) and a mean of €77 581 (99% CI: €46 910–€118 558) for behavioural and emotional disorders. We used the median costs in the base-case analysis and the lower bound of the median and upper bound of the mean as extreme values in the sensitivity analysis. We computed lifetime discounted costs based on the estimated LE for neurological sequelae.

## Results

### Base-case analysis

The model projected that the introduction of MCADD screening into the French newborn screening programme would, every year, prevent five child deaths and the occurrence of severe neurological sequelae in one child and mild neurological sequelae in one other child under five years, which results in a gain of 128 LY or 138 QALY (Table 2). The cost of the tests (including screening and confirmation tests) was estimated at €3.2 million. When the costs of treatment and care were taken into account, the net incremental cost of introducing MCADD screening was lower – estimated at €2.5 million – because of the MCADD complications prevented. The resulting ICER was €19 478 per LY or €18 033 per QALY gained.

**Table 2:** Cost-effectiveness of the introduction of MCADD newborn screening in France

**Description of results**	**Introduction of MCADD screening**	Introduction of MCADD screening combined with switch to MS/MS for PKU screening*
**Effectiveness**		
Deaths averted	5.47	5.47
Mild neurological sequelae prevented	1.37	1.37
Severe neurological sequelae prevented	1.37	1.37
LY gained	128	128
QALY gained	138	138
**Costs**		
Costs of testing alone^†^ (€)	3 187 660	1 742 702
Net incremental costs of screening ^‡^(€)	2 493 055	1 048 097
**Incremental cost-effectiveness ratios**		
€ per LY gained	19 478	8 189
€ per QALY gained	18 033	7 581

* The cost of the current technology PKU screening test (€1.76) was subtracted from that of the MS/MS screening test. The incremental effectiveness was the same as that for the introduction of MCADD screening alone as it was assumed that the performance of MS/MS for PKU screening was similar to that of the current technology; see text.

^†^ Includes the cost of screening and confirmation tests.

‡ Includes the cost of testing as well as the cost of follow-up and of management of diagnosed patients.

When the introduction of MCADD screening was combined with a switch in technology for PKU screening from fluorometry to MS/MS, the health gains remained the same as in the above strategy because the performances of MS/MS and of fluorometry for PKU screening were assumed to be similar. However, the incremental cost of the screening programme was lower because of the savings from no longer having to perform the current PKU test. The annual cost of the tests was estimated at €1.7 million and the annual net incremental cost of the screening programme at €1.0 million. The resulting ICER was estimated at €8 189 per LY or €7 851 per QALY gained. As expected, this strategy clearly dominated that of solely introducing MCADD screening.

### Sensitivity analyses

**Figure 2:**
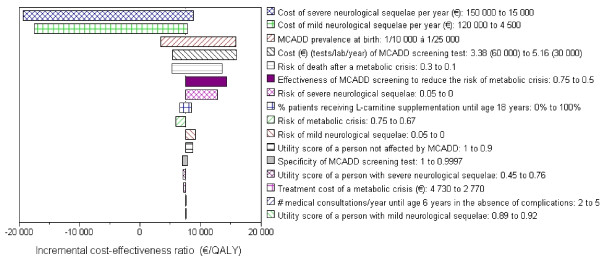
**Sensitivity analyses: Expansion of newborn screening to include MCADD*vs.*current newborn screening.** The width of the bar indicates the variation in the incremental cost-effectiveness ratio associated with alternative parameter values for that input. The numbers on the right side, next to the parameters, indicate the lower- and upper-bounds of the ranges used in sensitivity analyses.

One-way sensitivity analyses showed the influence of variations, within plausible ranges, of different parameters on the results of the model (Table 3 and Figure2). The parameters that had the largest influence include the costs of neurological sequelae, for which estimates were highly uncertain; the MCADD prevalence; the cost of the MCADD screening test, which is dependent on the number of tests per lab per year and ultimately on the number of labs performing screening tests; the risk of death following a metabolic crisis; and the effectiveness of MCADD screening. For example, the ICER varied from €3 444 to €15 856 per QALY gained when the MCADD prevalence varied from 1/1000 to 1/25 000. Likewise, the ICER rose to €15 655 per QALY if the cost per test was increased to €5.16 (which would correspond to 30 000 tests per lab per year). When the annual costs of managing neurological sequelae exceeded €50 000 for severe sequelae or €40 000 for mild sequelae, the programme became cost-saving.

**Table 3:** One-way sensitivity analyses of the cost-effectiveness of introducing MCADD screening and of switching to MS/MS technology for PKU screening

**Parameter**	**Value or range for sensitivity analyses**	**ICER (€/QALY)**
Base-case values		7 581
MCADD birth prevalence	1/10 000 to 1/25 000	3 444 to 15 856
MCADD screening test specificity	0.9997 to 1	7 878 to 6 987
Risk of developing a metabolic crisis	0.75	5 881
Risk of death within 72 hours of life	0.05	5 902
Risk of death after a metabolic crisis	0.01 to 0.03	13 180 to 5 314
Risk of mild neurological sequelae	0	9 175
Risk of severe neurological sequelae	0	12 823
Screening effectiveness (reduction in the risk of developing a metabolic crisis)	0.5	14 351
Utility of persons unaffected by MCADD	0.9	8 769
Utility of persons with severe neurological sequelae	0.45	7 121
Utility of persons with mild neurological sequelae	0.92	7 632
Cost of the MCADD screening test (€)	3.38 to 5.16	5 384 to 15 655
Annual cost of management of severe	15 000 to 150 000	8 832 to −19 139^*^
Annual cost of management of mild neurological sequelae (€)	4 500 to 120 000	7 911 to −17 353*
Cost of treatment of a metabolic crisis	4 730	7 211
% patients receiving L-carnitine supplementation until 18 years of age	0% to 100%	6 617 to 8 546
Number of medical consultations per year until 6 years of age	5	7 667
	No discounting	−514^*^
Annual discounting rate	3% to 6%	4 954 to 13 598

* A negative cost-effectiveness ratio indicates that the strategy is both more effective and less costly than the comparison strategy.

The worst-case scenario indicated that the ICER of introducing MCADD screening and of switching to MS/MS for PKU screening would be, at worst, equal to €72 115 per QALY gained.

## Discussion

The objective of this cost-effectiveness analysis was to inform policy makers and help them decide whether or not to introduce MCADD screening in France.

The predicted ICER of MCADD screening in France – €7 580 per QALY gained – is within the range of those obtained by others [10,13-15,18-23]. While there is no defined threshold in France for judging whether the implementation of a health intervention is an efficient allocation of public resources, this analysis suggests, in view of efficiency thresholds defined in other European countries, that the introduction of MCADD screening in France is cost-effective. In England, interventions with an ICER of less than £20 000 to £30 000 (around €24 000 to €36 000) per QALY gained are considered to be cost-effective [57]. In Sweden, the threshold to decide whether a drug provides value for money and to determine coverage status is €45 000 per QALY [58] and in the Netherlands, this threshold may be as high as €80 000 per QALY for severe conditions [59]. The World Health Organisation considers that health intervention are very cost-effective if they cost less than the average per capita gross domestic product (GDP) per disability adjusted life year averted (DALY) for a given country or region. Interventions that costs up to three times the average per capita GDP per DALY averted are still considered cost-effective, while those that exceed this threshold are considered not cost-effective [60]. For France, these thresholds would correspond to about €30 000 and €90 000 per DALY averted, respectively.

The results of the model were relatively robust to the variations of parameters applied in the one-way sensitivity analyses, whereby the ICER remains below €16 000 per QALY gained. The model was, however, more sensitive to some parameters, some of which are subject to a large degree of variability (notably the cost of neurological sequelae) or uncertainty (e.g. MCADD prevalence, cost of MCADD screening test). The production of empirical data generated by the implementation of MCADD screening in France would improve the estimates of some of these parameters.

The costs used in this analysis did not include the start-up costs for launching the programme, such as the costs of staff training and of producing information and education materials. While the use of MS/MS technology for newborn screening implies that the number of labs performing screening tests be reduced, the intangible costs related to potential disruption of the current newborn screening programme incurred by such reorganisation have not been taken into account either. We did not incorporate the potential loss of quality of life associated with lifelong dietary recommendations for treating MCADD in patients who may in the absence of screening not have experienced any MCADD related problems. Yet, such adjustments may counterbalance some of the health gains from newborn screening [23].

The budget impact for the national health insurance scheme of the expansion of the newborn screening programme to MCADD and of the replacement of existing technology for PKU screening was estimated at €1.7 million per year for the tests alone and at €1 million when the savings from preventing MCADD sequelae were taken into account. This amount represents an increase of 11% in the annual cost (currently estimated at €9 million) of the newborn screening programme.

## Conclusions

This analysis suggests that implementing MCADD newborn screening in France would be an efficient use of resources. These results were used by HAS as supporting evidence to recommend the expansion of newborn screening to include MCADD. The French Ministry of Health is currently making plans to put these public health recommendations into practice.

## Competing interests

The authors declare that they have no competing interests.

## Authors’ contributions

FFH conceived and designed the study and wrote the paper. CRP contributed to the conception of the study and provided overall advice. Both authors read and approved the final manuscript.

## References

[B1] Sander S Janzen N Janetzky B Scholl S Steuerwald U Schafer J Sander J Neonatal screening for medium chain acyl-CoA deficiency: high incidence in Lower Saxony (northern Germany) Eur J Pediatr 2001 160 5 318 319 11388605 10.1007/pl00008439

[B2] Schulze A Lindner M Kohlmuller D Olgemoller K Mayatepek E Hoffmann GF Expanded newborn screening for inborn errors of metabolism by electrospray ionization-tandem mass spectrometry: results, outcome, and implications Pediatrics 2003 111 6 Pt 1 1399 1406 12777559 10.1542/peds.111.6.1399

[B3] Maier EM Liebl B Roschinger W Nennstiel-Ratzel U Fingerhut R Olgemoller B Busch U Krone N v Kries R Roscher AA Population spectrum of ACADM genotypes correlated to biochemical phenotypes in newborn screening for medium-chain acyl-CoA dehydrogenase deficiency Hum Mutat 2005 25 5 443 452 15832312 10.1002/humu.20163

[B4] Derks TG Boer TS van Assen A Bos T Ruiter J Waterham HR Niezen-Koning KE Wanders RJ Rondeel JM Loeber JG Neonatal screening for medium-chain acyl-CoA dehydrogenase (MCAD) deficiency in The Netherlands: the importance of enzyme analysis to ascertain true MCAD deficiency J Inherit Metab Dis 2008 31 1 88 96 18188679 10.1007/s10545-007-0492-3

[B5] la Marca G Malvagia S Casetta B Pasquini E Donati MA Zammarchi E Progress in expanded newborn screening for metabolic conditions by LC- MS/MS in Tuscany: update on methods to reduce false tests J Inherit Metab Dis 2008 31 Suppl 2 S395 S404 18956250 10.1007/s10545-008-0965-z

[B6] Kasper DC Ratschmann R Metz TF Mechtler TP Möslinger D Konstantopoulou V Item CB Pollak A Herkner KR The national austrian newborn screening program - eight years experience with mass spectrometry. past, present, and future goals Wien Klin Wochenschr 2010 122 607 613 20938748 10.1007/s00508-010-1457-3

[B7] Shortland G Besmey J Newborn screening for medium-chain acyl-CoA dehydrogenase defociency (MCADD): findings from a multicentre prospective UK Collaborative study (abstract) J Inherit Metab Dis 2010 29 Suppl 1 19

[B8] Vilarinho L Rocha H Sousa C Marcao A Fonseca H Bogas M Osorio RV Four years of expanded newborn screening in Portugal with tandem mass spectrometry J Inherit Metab Dis 2010 10.1007/s10545-010-9048-z 20177789

[B9] Bodamer OA Hoffmann GF Lindner M Expanded newborn screening in Europe 2007 J Inherit Metab Dis 2007 30 4 439 444 17643197 10.1007/s10545-007-0666-z

[B10] Pollitt RJ Green A McCabe CJ Booth A Cooper NJ Leonard JV Nicholl J Nicholson P Tunaley JR Virdi NK Neonatal screening for inborn errors of metabolism: cost, yield and outcome Health Technol Assess 1997 1 7 9483160

[B11] Seymour CA Thomason MJ Chalmers RA Addison GM Bain MD Cockburn F Littlejohns P Lord J Wilcox AH Newborn screening for inborn errors of metabolism: a systematic review Health Technol Assess 1997 1 11 9483156

[B12] Medical Advisory Secretariat Neonatal screening of inborn errors of metabolism using tandem mass spectrometry: an evidence-based analysis Ontario Heath Technology Assessment Series 2003 3 3 PMC3387775 23074443

[B13] Pandor A Eastham J Beverley C Chilcott J Paisley S Clinical effectiveness and costeffectiveness of neonatal screening for inborn errors of metabolism using tandem mass spectrometry: a systematic eview (+annexes) Health Technol Assess 2004 8 12 10.3310/hta8120 14982654

[B14] Autti-Ramo I Makela M Sintonen H Koskinen H Laajalahti L Halila R Kaariainen H Lapatto R Nanto-Salonen K Pulkki K Expanding screening for rare metabolic disease in the newborn: an analysis of costs, effect and ethical consequences for decision-making in Finland Acta Paediatr 2005 94 8 1126 1136 16188860 10.1111/j.1651-2227.2005.tb02056.x

[B15] van der Hilst CS Derks TG Reijngoud DJ Smit GP TenVergert EM Cost- effectiveness of neonatal screening for medium chain acyl-CoA dehydrogenase deficiency: the homogeneous population of The Netherlands J Pediatr 2007 151 2 115 120 120 17643759 10.1016/j.jpeds.2007.03.013

[B16] Agence d’évaluation des technologies et des modes d’intervention en santé Spectrométrie de masse en tandem et dépistage des erreurs innées du métabolisme. Rapport technique 2007 AETMIS, Montreal

[B17] Wilcken B Haas M Joy P Wiley V Chaplin M Black C Fletcher J McGill J Boneh A Outcome of neonatal screening for medium-chain acyl-CoA dehydrogenase deficiency in Australia: a cohort study Lancet 2007 369 9555 37 42 17208640 10.1016/S0140-6736(07)60029-4

[B18] Schoen EJ Baker JC Colby CJ To TT Cost-benefit analysis of universal tandem mass spectrometry for newborn screening Pediatrics 2002 110 4 781 786 12359795 10.1542/peds.110.4.781

[B19] Insinga RP Laessig RH Hoffman GL Newborn screening with tandem mass spectrometry: examining its cost-effectiveness in the Wisconsin Newborn Screening Panel J Pediatr 2002 141 4 524 531 12378192 10.1067/mpd.2002.128116

[B20] Venditti LN Venditti CP Berry GT Kaplan PB Kaye EM Glick H Stanley CA Newborn screening by tandem mass spectrometry for medium-chain Acyl-CoA dehydrogenase deficiency: a cost-effectiveness analysis Pediatrics 2003 112 5 1005 1015 14595039 10.1542/peds.112.5.1005

[B21] Tran K Banerjee S Li H Noorani HZ Mensinkai S Dooley K Clinical efficacy and cost-effectiveness of newborn screening for medium chain acyl-CoA dehydrogenase deficiency using tandem mass spectrometry Clin Biochem 2007 40 3–4 235 241 17222812 10.1016/j.clinbiochem.2006.10.022

[B22] Cipriano LE Rupar CA Zaric GS The cost-effectiveness of expanding newborn screening for up to 21 inherited metabolic disorders using tandem mass spectrometry: results from a decision-analytic model Value Health 2007 10 2 83 97 17391418 10.1111/j.1524-4733.2006.00156.x

[B23] Prosser LA Kong CY Rusinak D Waisbren SL Projected costs, risks, and benefits of expanded newborn screening for MCADD Pediatrics 2010 125 2 e286 e294 20123779 10.1542/peds.2009-0605

[B24] Norman R Haas M Wilcken B International perspectives on the cost- effectiveness of tandem mass spectrometry for rare metabolic conditions Health Policy 2009 89 3 252 260 18823674 10.1016/j.healthpol.2008.08.003

[B25] Grosse SD Baily MA, Murray TH Cost effectiveness as a criterion for newborn screening policy decisions Ethics and newborn genetic screening: new technologies, new challenges 2009 Johns Hopkins University Press, Baltimore 58 88

[B26] Haute Autorité de Santé Choix méthodologiques pour l’évaluation éconmique à la HAS 2011 HAS, Saint-Denis La Plaine

[B27] Haute Autorité de Santé Evaluation a priori de l’extension du dépistage néonatal à une ou plusieurs erreurs innées du métabolisme par la technique de spectrométrie de masse en tandem en population générale en France. 1er volet: dépistage du déficit en MCAD 2011 HAS, Saint-Denis La Plaine

[B28] Wilcken B Wiley V Hammond J Carpenter K Screening newborns for inborn errors of metabolism by tandem mass spectrometry N Engl J Med 2003 348 23 2304 2312 12788994 10.1056/NEJMoa025225

[B29] Chace DH Hillman SL Van Hove JL Naylor EW Rapid diagnosis of MCAD deficiency: quantitative analysis of octanoylcarnitine and other acylcarnitines in newborn blood spots by tandem mass spectrometry Clin Chem 1997 43 11 2106 2113 9365395

[B30] Carpenter K Wiley V Sim KG Heath D Wilcken B Evaluation of newborn screening for medium chain acyl-CoA dehydrogenase deficiency in 275 000 babies Arch Dis Child Fetal Neonatal Ed 2001 85 2 F105 F109 11517203 10.1136/fn.85.2.F105 PMC1721303

[B31] Pourfarzam M Morris A Appleton M Craft A Bartlett K Neonatal screening for medium-chain acyl-CoA dehydrogenase deficiency Lancet 2001 358 9287 1063 1064 11589939 10.1016/S0140-6736(01)06199-2

[B32] Zytkovicz TH Fitzgerald EF Marsden D Larson CA Shih VE Johnson DM Strauss AW Comeau AM Eaton RB Grady GF Tandem mass spectrometric analysis for amino, organic, and fatty acid disorders in newborn dried blood spots: a two-year summary from the New England Newborn Screening Program Clin Chem 2001 47 11 1945 1955 11673361

[B33] Frazier DM Millington DS McCandless SE Koeberl DD Weavil SD Chaing SH Muenzer J The tandem mass spectrometry newborn screening experience in North Carolina: 1997–2005 J Inherit Metab Dis 2006 29 1 76 85 16601872 10.1007/s10545-006-0228-9

[B34] Feuchtbaum L Lorey F Faulkner L Sherwin J Currier R Bhandal A Cunningham G California’s experience implementing a pilot newborn supplemental screening program using tandem mass spectrometry Pediatrics 2006 117 5 Pt 2 S261 S269 16735252 10.1542/peds.2005-2633E

[B35] Hsu HW Zytkovicz TH Comeau AM Strauss AW Marsden D Shih VE Grady GF Eaton RB Spectrum of medium-chain acyl-CoA dehydrogenase deficiency detected by newborn screening Pediatrics 2008 121 5 e1108 e1114 18450854 10.1542/peds.2007-1993

[B36] Horvath GA Davidson AG Stockler-Ipsiroglu SG Lillquist YP Waters PJ Olpin S Andresen BS Palaty J Nelson J Vallance H Newborn screening for MCAD deficiency: experience of the first three years in British Columbia, Canada Can J Public Health 2008 99 4 276 280 18767270 10.1007/BF03403754 PMC6975828

[B37] Grosse SD Khoury MJ Greene CL Crider KS Pollitt RJ The epidemiology of medium chain acyl-CoA dehydrogenase deficiency: an update Genet Med 2006 8 4 205 212 16617240 10.1097/01.gim.0000204472.25153.8d

[B38] Touma EH Charpentier C Medium chain acyl-CoA dehydrogenase deficiency Arch Dis Child 1992 67 1 142 145 1739332 10.1136/adc.67.1.142 PMC1793557

[B39] Wilcken B Hammond J Silink M Morbidity and mortality in medium chain acyl coenzyme A dehydrogenase deficiency Arch Dis Child 1994 70 5 410 412 8017963 10.1136/adc.70.5.410 PMC1029830

[B40] Iafolla AK Thompson RJ Roe CR Medium-chain acyl-coenzyme A dehydrogenase deficiency: clinical course in 120 affected children J Pediatr 1994 124 3 409 415 8120710 10.1016/s0022-3476(94)70363-9

[B41] Pollitt RJ Leonard JV Prospective surveillance study of medium chain acyl-CoA dehydrogenase deficiency in the UK Arch Dis Child 1998 79 2 116 119 9797590 10.1136/adc.79.2.116 PMC1717653

[B42] Klose DA Kolker S Heinrich B Prietsch V Mayatepek E von Kries R Hoffmann GF Incidence and short-term outcome of children with symptomatic presentation of organic acid and fatty acid oxidation disorders in Germany Pediatrics 2002 110 6 1204 1211 12456920 10.1542/peds.110.6.1204

[B43] Derks TG Reijngoud DJ Waterham HR Gerver WJ van den Berg MP Sauer PJ Smit GP The natural history of medium-chain acyl CoA dehydrogenase deficiency in the Netherlands: clinical presentation and outcome J Pediatr 2006 148 5 665 670 16737882 10.1016/j.jpeds.2005.12.028

[B44] Wilcken B Haas M Joy P Wiley V Bowling F Carpenter K Christodoulou J Cowley D Ellaway C Fletcher J Expanded newborn screening: outcome in screened and unscreened patients at age 6 years Pediatrics 2009 124 2 e241 e248 19620191 10.1542/peds.2008-0586

[B45] Waisbren SE Albers S Amato S Ampola M Brewster TG Demmer L Eaton RB Greenstein R Korson M Larson C Effect of expanded newborn screening for biochemical genetic disorders on child outcomes and parental stress JAMA 2003 290 19 2564 2572 14625333 10.1001/jama.290.19.2564

[B46] Dezateux C Newborn screening for medium chain acyl-CoA dehydrogenase deficiency: evaluating the effects on outcome Eur J Pediatr 2003 162 Suppl 1 S25 S28 14628139 10.1007/s00431-003-1346-0

[B47] Strauss D Eyman RK Mortality of people with mental retardation in California with and without Down syndrome, 1986–1991 Am J Ment Retard 1996 100 6 643 653 8735577

[B48] Abadie V Berthelot J Feillet F Maurin N Mercier A Ogier de Baulny H de Parscau L Neonatal screening and long-term follow-up of phenylketonuria: the French database Early Hum Dev 2001 65 149 158 11641035 10.1016/s0378-3782(01)00223-7

[B49] Chace DH Sherwin JE Hillman SL Lorey F Cunningham GC Use of phenylalanine-to-tyrosine ratio determined by tandem mass spectrometry to improve newborn screening for phenylketonuria of early discharge specimens collected in the first 24 hours Clin Chem 1998 44 12 2405 2409 9836704

[B50] Ceglarek U Muller P Stach B Buhrdel P Thiery J Kiess W Validation of the phenylalanine/tyrosine ratio determined by tandem mass spectrometry: sensitive newborn screening for phenylketonuria Clin Chem Lab Med 2002 40 7 693 697 12241016 10.1515/CCLM.2002.119

[B51] Wilcken B Recent advances in newborn screening J Inherit Metab Dis 2007 30 2 129 133 17342450 10.1007/s10545-007-0538-6

[B52] Wilcken B More on medium-chain acyl-coenzyme a dehydrogenase deficiency in a neonate N Engl J Med 2008 358 6 647 18256406 10.1056/NEJMc073220

[B53] Bras MA Pégaz-Blanc O Di Franco M Kocoglu D Martial F Roosz P Tronyo J Cotis JP de Peretti G Tableaux de l’économie française 2010 Institut national de la statistique et des études économiques, In. Paris

[B54] Prosser LA Ladapo JA Rusinak D Waisbren SE Parental tolerance of false-positive newborn screening results Arch Pediatr Adolesc Med 2008 162 9 870 876 18762606 10.1001/archpediatrics.2008.1

[B55] Association française pour le dépistage et la prévention des handicaps de l’enfant Bilan d’activité 2009 AFDPHE, Paris

[B56] Nasser M Javaheri H Fedorowicz Z Noorani Z Carnitine supplementation for inborn errors of metabolism Cochrane Database Syst Rev 2009 2 10.1002/14651858.CD006659.pub2 19370646

[B57] National Institute for Health and Clinical Excellence Social value judgements. Principles for the development of NICE guidance 2008 NICE, London 27905706

[B58] Sorenson C The role of HTA in coverage and pricing decisions: A cross- country comparison Euro Observer 2009 11 1 1 4

[B59] Poley MJ Stolk EA Brouwer WBF The use and impact of HTA in decision making in the Netherlands Euro Observer 2009 11 1 7 8

[B60] WHO Commission on Macroeconomics and Health Macroeconomics and health: investing in health for economic development 2001 World Health Organization, Geneva

